# Early Diagnostics and Early Intervention in Neurodevelopmental Disorders—Age-Dependent Challenges and Opportunities

**DOI:** 10.3390/jcm10040861

**Published:** 2021-02-19

**Authors:** Mijna Hadders-Algra

**Affiliations:** University of Groningen, University Medical Center Groningen, Department of Paediatrics—Section Developmental Neurology, 9713 GZ Groningen, The Netherlands; m.hadders-algra@umcg.nl; Tel.: +31-50-3614247

**Keywords:** cerebral palsy, intellectual disability, autism spectrum disorder, early detection, early intervention, family, magnetic resonance imaging, general movement assessment, cortical subplate

## Abstract

This review discusses early diagnostics and early intervention in developmental disorders in the light of brain development. The best instruments for early detection of cerebral palsy (CP) with or without intellectual disability are neonatal magnetic resonance imaging, general movements assessment at 2–4 months and from 2–4 months onwards, the Hammersmith Infant Neurological Examination and Standardized Infant NeuroDevelopmental Assessment. Early detection of autism spectrum disorders (ASD) is difficult; its first signs emerge at the end of the first year. Prediction with the Modified Checklist for Autism in Toddlers and Infant Toddler Checklist is possible to some extent and improves during the second year, especially in children at familial risk of ASD. Thus, prediction improves substantially when transient brain structures have been replaced by permanent circuitries. At around 3 months the cortical subplate has dissolved in primary motor and sensory cortices; around 12 months the cortical subplate in prefrontal and parieto-temporal cortices and cerebellar external granular layer have disappeared. This review stresses that families are pivotal in early intervention. It summarizes evidence on the effectiveness of early intervention in medically fragile neonates, infants at low to moderate risk, infants with or at high risk of CP and with or at high risk of ASD.

## 1. Introduction

Many children have a neurodevelopmental disorder, such as cerebral palsy (CP), intellectual disability or autism spectrum disorder (ASD). In high income countries the prevalence of CP is 1–3‰ [1,2,3], that of intellectual disability is about 1% [2,4] and that reported for ASD is 0.5–3% [5,6]. In low income countries, relatively little information on the prevalence of neurodevelopmental disorders is available. The limited data suggest that the prevalence has been increasing during the last two decades [7]. They also indicate that the prevalence of CP is at the high end of the prevalence range in high income countries [8,9], whereas the prevalence of intellectual disability is higher and that of ASD is lower than that in high income countries [10]. The latter is presumably due to underdiagnostics, as suggested by the prevalence data of Taiwan, which indicated that improved diagnostics were associated with a five-fold increase in the prevalence of ASD between 2000 and 2011 [6].

Neurodevelopmental disorders form a heterogeneous group of disorders. Also, the diagnostic entities themselves comprise a broad spectrum of clinical presentations. Yet what the disorders share is their early neurodevelopmental origin. Factors early in life, either genetic, environmental or both, interrupt the complex sequences involved in typical brain development. This is reflected by the neural substrate of the disorders documented in neuroimaging studies (performed in individuals who have reached at least school-age)—the disorders are characterized by widespread alterations of the brain’s structure, in which the abnormalities are just as heterogeneous as the clinical phenotypes. The widespread deviancies are found in the brain’s grey and white matter [11,12,13]. Nonetheless, the neural network anomalies have some disorder-specific characteristics. For instance, the network anomalies reported in children with CP virtually always include lesions of the white matter of the sensorimotor areas, often comprising the periventricular white matter [11,14], whereas those reported in children with ASD virtually always include abnormalities in the networks of the so-called ‘social brain’, that comprise the fronto-temporal and fronto-parietal regions [13,15].

The early origin of the neurodevelopmental disorders would potentially allow their early detection and hence an early onset of intervention, that is, intervention in a time window characterized by high neural plasticity. However, the rapid developmental changes underlying neural plasticity interfere with early detection. It takes developmental time before signs of specific disorders emerge. The primary aim of this paper is to review current knowledge on the opportunities and challenges in early detection of developmental disorders. Focus is on the first two post-natal years, with special emphasis on the first year post-term. The review’s body on the early detection of developmental disorders (Section 3) is preceded by a summary of the developmental changes in the brain in early life (Section 2). It is followed (in Section 4) by a concise review of early intervention approaches used in young children with or at high risk of developmental disorders (for details see reference [16]).

The paper concludes that neonatal magnetic resonance imaging (MRI) is certainly helpful in the prediction of developmental outcome in terms of CP with or without intellectual disability. Yet, neonatal MRI is not available for the large majority of children. In these children we have to rely on neurodevelopmental and neurobehavioral assessments and questionnaires. Prediction of CP with or without intellectual disability with neurodevelopmental assessments improves substantially from 2–4 months corrected age (CA) onwards. Early prediction of ASD is difficult; its first signs emerge at the end of the first year. The review suggests that this means that the prediction of developmental disorders improves substantially when the transient structures of the young brain have been taken over by permanent circuitries. One transition is around 3 months, when the cortical subplate in the primary motor and sensory cortices has dissolved—hence the improved prediction of CP with or without intellectual disability from this age onwards. A second transition occurs around 12 months when the cortical subplate in the prefrontal and parieto-temporal cortices and the external granular layer in the cerebellum have disappeared. After this transition, the prediction of ASD becomes possible.

## 2. Early Human Brain Development

**Figure 1:**
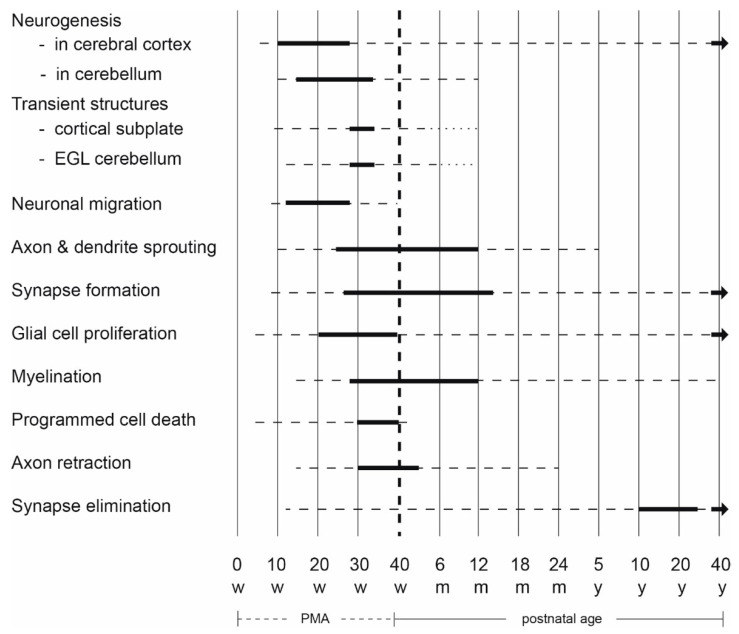
Schematic overview of the developmental processes occurring in the human brain. The bold lines indicate that the processes mentioned on the left side are very active, the broken lines denote that the processes still continue but less abundantly. The diagram is based on reference [17]. EGL = external granular layer; m = months; PMA = postmenstrual age; w = weeks; y = years. Figure reproduced with permission from ‘Early Detection and Early Intervention in Developmental Motor Disorders—from neuroscience to participation’ by Mijna Hadders-Algra (ed.) published by Mac Keith Press in its Clinics in Developmental Medicine Series, ISBN number 978-1-911612-43-8 [16].

It takes the human brain about forty years to reach its full-blown adult configuration. The developmental processes involved are the result of an intricate continuous interaction between genes and environment, activity and experience [17]. The processes that occur during early development are summarized in Figure 1; details are provided in reference [17].

The development of the nervous system starts with the formation of the neural tube in the fifth week postmenstrual age (PMA). Shortly after closure of the neural tube, specific areas near the ventricles start to generate neurons. The majority of cerebral neurons are generated between 5 and 28 weeks PMA in the germinal layers near the ventricles [18,19,20]. From their site of origin in the germinal layers, neurons migrate radially or tangentially to their places of destination [21]. The destination site of many cortical neurons is located in the more superficially situated cortical plate. The process of migration peaks between 20 and 26 weeks PMA, with a minor spatio-temporal gradient implying that the migratory peak in the occipital regions occurs slightly earlier than that in the frontal areas [22]. During migration, the neurons start to differentiate, that is, they form axons, dendrites, synapses with transmitters and receptors, the intracellular machinery and the complex neuronal membranes. Interestingly, the first generations of neurons do not reach the cortical plate but they halt in the cortical subplate.

The cortical subplate is a temporary structure between the cortical plate and the future white matter. It is a hotspot of brain development during fetal life—it is the major site of neuronal differentiation and synaptogenesis and it receives the first in-growing cortical afferents (e.g., the thalamocortical afferents) [20]. Most information on the subplate is based on animal research; relatively little is known on the subplate’s developmental details and its exact function during human development [23]. But we do know the following; during mid-gestation the subplate is the main site of synaptic activity in the brain and a major mediator of fetal behavior [20]. The subplate is thickest at 28–34 weeks PMA. At that time it is four to seven times thicker than the cortical plate, with the largest relative size occurring in the frontal and parietal association areas [24,25]. From 25–26 weeks onwards, the subplate starts to shrink gradually, as its neurons die due to programmed cell death. In this phase, later generated neurons pass through the decreasing subplate and start to populate the cortical plate. These developmental changes comprise a relocation of the thalamocortical afferents that now grow to their final targets in the cortical plate [20,24].

The expansion of the cortical plate is associated with an increase of the cortex and the onset of gyrification. Estimates suggest that cortical volume increases about twenty-fold in the second half of gestation. This increase coincides with the decrease of the cortical subplate. This implies that the human cortex during the third trimester of gestation and the early post-term period is characterized by the co-existence of two separate but interconnected cortical circuitries—the transient circuitries of the subplate and the developing permanent circuitries in the cortical plate. The situation with the double circuitries ends when the subplate has dissolved. This occurs at around 3 months post-term in the primary motor, somatosensory and visual cortices and around 1 year in the cortical association areas including the prefrontal and the parieto-temporal regions [25,26].

Brain development also comprises the generation of glial cells. Ultimately the adult brain contains approximately 85 billion glial cells (and about an equal number of neurons) [27]. Glial cells are generated particularly during the second half of gestation. A specific subgroup of glial cells, the oligodendrocytes, is in charge of axonal myelination. The oligodendrocytes develop especially between 28 and 40 weeks PMA, a process that is accompanied by rapid myelination. The intensive myelination continues during the first 6 months post-term [28,29]. Thereafter, many years of myelination follow, as myelination of the cortex is first completed around the age of 40 years [30].

Brain development not only involves processes of generation and synthesis, it also includes regressive phenomena. The process of neuronal death has already been mentioned. Animal research indicates that about half of the created neurons die off through apoptosis [31]. Apoptotic cell death is the result of interaction between endogenous programs and the chemical and electrical signals induced by experience. Other regressive processes are axon and synapse elimination. In humans, axon elimination has been described best for the corpus callosum and the corticospinal tract. Retraction of callosal axons occurs especially in the third trimester of gestation and the first two months post-term [32]. Axon elimination in the corticospinal tract starts in the third trimester of gestation and continues during the first two postnatal years. It transforms the corticospinal tract from an initially bilateral fiber system into a predominantly contralateral projection [33]. This reorganization is activity driven and use dependent. This may be illustrated by the effect of an early unilateral lesion of the brain inducing asymmetrical activity in the spinal cord. This asymmetrical activity results in a preferential strengthening of the ipsilateral projections of the contra-lesional hemisphere in comparison to the contralateral projections of the ipsi-lesional hemisphere [34]. Finally, the elimination of synapses in the brain starts during mid-gestation. Yet, cortical synapse elimination is particularly prominent between the onset of puberty and early adulthood [35].

From early age onwards, transmitters and receptors form part of the neural tissue. Already at 8–10 weeks, PMA catecholamines, serotonin, γ-aminobutyric acid (GABA) and excitatory amino acids including glutamate are found in the cerebral cortex [36]. In the development of the neurotransmitter systems the peri-term period is a distinct phase [36]. During this time window the noradrenergic α2- receptors in the brain’s white matter and many brain stem nuclei are transiently overexpressed and dopamine’s turnover is relatively high. Also, the development of glutamatergic *N*-methyl-D-aspartate (NMDA) receptors in the brain is characterized by transient overexpression. This overexpression occurs twice—first during early gestation (between 13 and 21 weeks PMA) and a second time around term age. Two additional changes occur in the peri-term period. In the third trimester GABA changes from having and excitatory function—which is characteristic of GABA in the first two trimesters—into having an inhibitory function, which is typical during the rest of life [36,37]. In addition, serotonergic innervation of the cortex rapidly changes after term age: at term the serotonergic fibers penetrate all cortical layers but they quickly decrease in density in the following few weeks [36]. It has been suggested that the specific neurotransmitter setting around term age induces a physiological, increased excitability that is, for instance, expressed in the motoneurons. Conceivably this setting serves postnatal survival by assisting the replacement of the fetal episodic breathing pattern by continuous respiration [38].

The development of the cerebellum, which hosts about 80% of the neurons of the adult brain [27], involves similar processes as described above but with its own time table. Cells in the cerebellum originate from two proliferative zones: (1) the ventricular zone, which brings forth the deep cerebellar nuclei and the Purkinje cells, and (2) the external granular layer originating from the rhombic lip [39,40]. Cell proliferation in the ventricular zone starts at 11 weeks and in the external granular layer at 15 weeks PMA. The external granular layer is a transient structure that generates the granule cells, the most numerous cells of the cerebellum. The layer is thickest between 28 and 34 weeks. From the external granular layer the granule cells migrate to their site of origin, most often the internal granular layer. The latter layer especially grows between mid-gestation and 3 months post-term. The external granular layer rapidly decreases in size between 2 and 3 months post-term, but it takes until the end of the first postnatal year before the transient external granular layer has disappeared [17,39].

In summary, during fetal life and the first two years postnatally the brain shows strong developmental activity. The peak of developmental activity occurs in the second half of gestation and the first three months post-term but developmental activity continues to be high in the first year post-term. High developmental activity implies high neuroplasticity, suggesting that especially the first year offers great opportunities for early intervention to improve the child’s developmental outcome. Interestingly, brain development involves the presence of transient structures. This means that behavior at early stages is more mediated by other circuitries (which include transient structures) than behavior at later ages, when the function of transient structures has been taken over by permanent circuitries. For diagnostics this implies that two age periods are relevant: (1) the period around 3 months post-term when the cortical subplate in the primary motor, somatosensory and visual cortices has dissolved, and (2) the end of the first year, when the cortical subplate in the cortical association areas and the cerebellar external granular layer have disappeared.

## 3. Early Detection of Developmental Disorders

This paragraph first discusses the implications of the developmental changes in the young brain for early detection of developmental disorders. Next, it reviews the most relevant clinical methods of early detection: (a) neuroimaging; (b) neurological assessments; (c) motor assessments; (d) developmental assessments; and (e) instruments aiming at early detection of ASD. This does not mean that other instruments such as neurophysiological assessments, including electroencephalography (EEG) and evoked potentials and sensory assessments, do not play a role in early diagnostics. These tools are certainly useful but generally, they are used on specific indication. For instance, continuous and amplitude-integrated EEG and somatosensory evoked potentials are applied in infants with hypoxic-ischemic encephalopathy (HIE) [41,42] and sensory assessments are used in preterm infants with behavioral issues [43,44]. Therefore, these instruments fall beyond the scope of this review.

### 3.1. Diagnostic Implications of the Developing Brain

The dynamic developmental changes occurring during the first two years postnatally have three clinical implications for the early detection of developmental disorders. First, assessments need to be age-specific, that is, the assessment techniques and assessment criteria should be adapted to the age-specific properties of the infant brain. Hence, a neurological assessment of a newborn comprises other items and has other criteria for typical and atypical performance than a neurological examination of a 12-month-old infant (cf. the Hammersmith Neonatal Neurological Examination [45] and the Hammersmith Infant Neurological Examination [46]). Likewise, the criteria to classify neuroimaging scans as typical or atypical are age-dependent [47].

Second, the developmental processes in the brain may induce changes in the infant’s neurodevelopmental performance. Infants with neurological deviancies in the first months post-term may recover and have a typical developmental outcome [48]. For instance, more than half of the children who had shown clear neurological dysfunction in the neonatal period had a typical neurodevelopmental outcome at 14 years [48]. Other infants show typical neurodevelopmental performance in early infancy but are diagnosed with a neurodevelopmental disorder later on (e.g., unilateral spastic CP or ASD [49,50]). This means that caution is needed in the prediction of developmental outcome at an early age. Yet, we do know that a combination of tools recommended in the child’s context with the infant’s clinical history and clinical signs does assist prediction. For instance, it has been well established that the combination of a neonatal MRI scan of the brain in combination with general movement assessment at 2–4 months corrected age (CA) in infants who have been admitted to the neonatal intensive care has high predictive power for CP [51,52]. Yet, the majority of infants with a developmental disorder do not have a history of neonatal intensive care. In these infants neonatal neuroimaging is lacking and general movement assessment has a good, but less robust, predictive validity [49].

Third, the way in which neurodevelopmental dysfunction is expressed changes with increasing infant age. Newborn infants, including infants with a unilateral brain lesion, express neurological deviancy virtually always by means of generalized signs, for example, they show generalized hypertonia, generalized hypotonia, a hyperexcitability syndrome or atypical general movements. If an infant with a unilateral brain lesion is later diagnosed with unilateral spastic CP—which happens in 35%–65% of the infants with this type of brain lesion (depending on etiology and timing [53,54])—the signs of asymmetry gradually emerge. Subtle asymmetries may be detected at 3–5 months when the general movements are replaced by goal directed movements [55]. With increasing age, the asymmetries become more pronounced, with the picture of unilateral CP becoming more prominent during the second half of the first year [56]. Conceivably, the emerging asymmetry is related to changes in the corticospinal tract developing from a bilateral projection system to a predominantly unilateral system.

A reliable assessment of the infant’s neurological, motor, developmental and behavioral status requires that the infant is in an adequate behavioral state. This means that the infant is not crying nor sucking on a pacifier, as this interferes with the test results [57]. During most tests infants also should not sleep. The exception to this rule is that general movements may be assessed during active sleep [57]. The latter is convenient information for the assessment of general movements around term age, when it is sometimes challenging to obtain a sufficiently long video-recording of an infant in an awake, non-crying behavioral state—due to the infant’s increased neurophysiological excitability at this age (see Section 2).

### 3.2. Neuroimaging

Studies on the predictive value of neuroimaging focused on neonatal assessments in infants with HIE and preterm infants. Currently, MRI is the gold standard in neonatal neuroimaging [58,59,60]. Nonetheless, in many places in the world neonatal MRI facilities are not available. In these situations, the bedside technique of cranial ultrasonography offers a good alternative [61].

Meta-analyses indicated that MRI is a useful tool to predict adverse outcome in infants with HIE born after a pregnancy of at least 35 weeks [41,42,62,63]. Prediction is equally good in infants who have or have not been treated with hypothermia [63] Abnormalities on conventional MRI scans during the first postnatal week predict adverse outcome (defined as the presence of moderate to severe neurological impairment or death) with a sensitivity of 85% and a specificity of 86%–89%. MRI scans made in the second to fourth week after birth are associated with a higher sensitivity (99%) but a lower specificity (53%) [62]. Overall prediction of an abnormal MRI scan during the first four weeks postnatally results in a sensitivity of 91% and a specificity of 51%. If only abnormalities in the posterior limb of the internal capsule (PLIC) are taken into account sensitivity decreases to 71% but specificity rises to 86% [62]. Best prediction during the first four postnatal weeks is achieved with magnetic resonance spectroscopy (MRS) of the thalamus and basal ganglia. An atypical lactate/*N*-acetylaspartate ratio predicts adverse outcome with a sensitivity of 82% and a specificity of 95% [62]. Abnormalities in the PLIC and in the thalamus and basal ganglia diagnosed by means of the advanced technique of diffusion-weighted imaging also strongly predict adverse outcome [42].

Meta-analysis of the prognostic value of conventional MRI in preterm infants born before 33 weeks of gestation indicated that in particular scans made at term equivalent age (TEA) predict outcome relatively well [64]. Abnormalities on MRI-scans at TEA predict CP with a sensitivity of 77% and a specificity of 79% but predict intellectual disability less appropriately (sensitivity 66%, specificity 61%) [64]. Lesions of the white matter predict neurodevelopmental impairment best [64]. However, this does not hold true for diffuse excessive high signal intensity (DESHI) in the periventricular and subcortical white matter, as it inaccurately reflects the connectivity’s integrity. Hence, it does not adequately predict CP and intellectual disability [65]. Moderate to severe abnormalities on MRI scans made before 36 weeks also predict neurodisability at one year [66]. It is conceivable that advanced MRI techniques, including diffusion-weighted imaging and MRS, may assist improved prediction of neurodevelopmental disorders but the limited information available does not yet allow for conclusions about their applicability in clinical practice [58,67,68,69].

Despite the fact that cranial ultrasound is no longer the gold standard of neonatal neuroimaging, it continues to play a role in the early diagnostics of brain lesions and developmental disorders in preterm infants. This is true for hospitals with and without neonatal MRI facilities, as ultrasonography is a bedside technique that can be easily repeated. The latter allows for sequential scans that may reveal developmental changes in white matter abnormalities [61]. The combination of sequential ultrasound scans and MRI at TEA results in a better prediction of outcome than that based on MRI at TEA alone [70].

### 3.3. Clinical Assessments

#### 3.3.1. Neurological Assessments

Standardized neurological assessments designed for the evaluation of the integrity of the nervous system of young children emerged in the second half of last century. Examples are the neonatal neurological examination of Prechtl [71], the Hammersmith Neonatal Neurological Examination (HNNE) [45], the Amiel-Tison Neurological Examination (ATNA) [72] and the Hammersmith Infant Neurological Examination (HINE) [46]. The methods reflect their time of origin; they focus on muscle tone, reflexes and reactions, and pay relatively little attention to the quality of spontaneous movements. In the last two decades of the 20th century it became clear that the nervous system is not a reactive system mainly organized in chains of reflexes, but a system primarily characterized by intrinsic, spontaneous activity [17,73]. This conceptual shift paved the way for the notion that neurological integrity is not only expressed in terms of tone and reflexes, but also—or rather, even more so—in the quality of the infant’s spontaneous movements [74,75] (see also Section 3.3.2). The recently developed neurological scale of the Standardized Infant NeuroDevelopmental Assessment (SINDA) followed these ideas by addressing in a substantial proportion of items the quality of spontaneous movements [76].

Of the neonatal examinations, HNNE’s use is most frequently reported in the literature (for details see reference [57]). It consists of 34 items that can be assessed in less than 15 min [45]. Its outcome is an optimality score of maximally 34 points. Local norms to define at risk scores are recommended [77,78], but only available to a limited extent. It is also advised to perform HNNE in preterm infants at TEA. Yet, the study of Venkata et al. [79] suggests that the predictive properties of HNNE performed around 36 weeks, that is, at an age that most preterm infants are still in the hospital, is similar to that of HNNE at TEA. The predictive values of HNNE in preterm infants vary. In very preterm infants HNNE scores were associated with a high negative, but a low positive predictive value to predict neurosensory impairment at 2 years [80]. In moderate-to-late preterm infants HNNE scores were associated with an increased risk of cognitive delay at 2 years, but not with motor and language outcome [77]. In a mixed group of infants mostly comprising preterm infants, HNNE predicted adverse neurodevelopmental outcome at 1 year with sensitivities of 50%–64% and specificities of 73%–77% [79]. A study performed in the pre-hypothermia era in infants with HIE indicated that HNNE has good predictive properties [81]. It is conceivable that the reported difference in the predictive properties of HNNE between preterm infants and infants with HIE can be attributed to the higher proportion of infants with a serious brain lesion in the latter than in the former study groups.

ATNA has primarily been developed to describe the current neurological condition of children aged 0 to 6 years. It is not aiming to predict developmental outcome [72,82]. Assessment of its maximally 41 items has good reliability and can be performed in 10–15 min. No studies have been performed on ATNA’s predictive properties in infants less than 1 year. ATNA’s classification of disabling impairment at 1 year predicted disabling impairment at 14–15 years with a sensitivity of 38% and a specificity of 98% [83]. In addition, a worse neurological condition at 2 years of age has been associated with lower IQ and a higher prevalence of learning disorders at school-age [84,85].

The HINE is the most frequently used infant neurological examination, designed for children up to 2 years of age [46,82]. It consists of 26 items, scored on a 4-point scale, that can be reliably assessed in less than 10 min. The cut-offs of the item scores and the criteria for ‘at risk’ are age dependent. The latter are only available for the ages of 3, 6, 9, 12 and 18 months. In clinical samples, the HINE ‘at risk’ score predicts CP with sensitivities of 90–100% and specificities of 85–100% [82] and intellectual disability with sensitivities of 51–82% and specificities of 71–90% [86]. A retrospective clinical study suggested that HINE’s asymmetry score may assist the prediction of unilateral spastic CP [87].

The neurological scale of the SINDA has been developed recently. It is designed for infants aged 6 weeks to 12 months. SINDA’s neurological scale consists of 28 dichotomous items that can be assessed reliably in less than 10 min [76]. Two studies in clinical samples reported that SINDA’s neurological scale has powerful predictive properties. It predicted developmental disorder (CP and/or intellectual disability) with sensitivities of 83–89% and specificities of 94–96% and CP with sensitivities of 91–100% and specificities 81–85% [76,88].

#### 3.3.2. Motor Assessments

This paragraph focusses on motor assessments that—by design—aim to predict developmental outcome. This means that assessments primarily aiming to discriminate between specific groups of infants and to monitor motor development, such as the Alberta Infant Motor Scale (AIMS) [89] and the Bayley Scales of Infant and Toddler Development (BSID) [90], are not discussed. The three motor assessments that are used to predict outcome are the general movement assessment (GMA) [91], the Test of Infant Motor Performance (TIMP) [92] and the Infant Motor Profile (IMP) [93]. Note that these test also are used for discrimination and monitoring. In addition, the IMP is a responsive instrument to evaluate the effect of early intervention [94,95,96,97]. The three predictive tests are partially (TIMP) or totally (GMA and IMP) based on the observation of the quality of self-generated movements.

**Figure 2:**
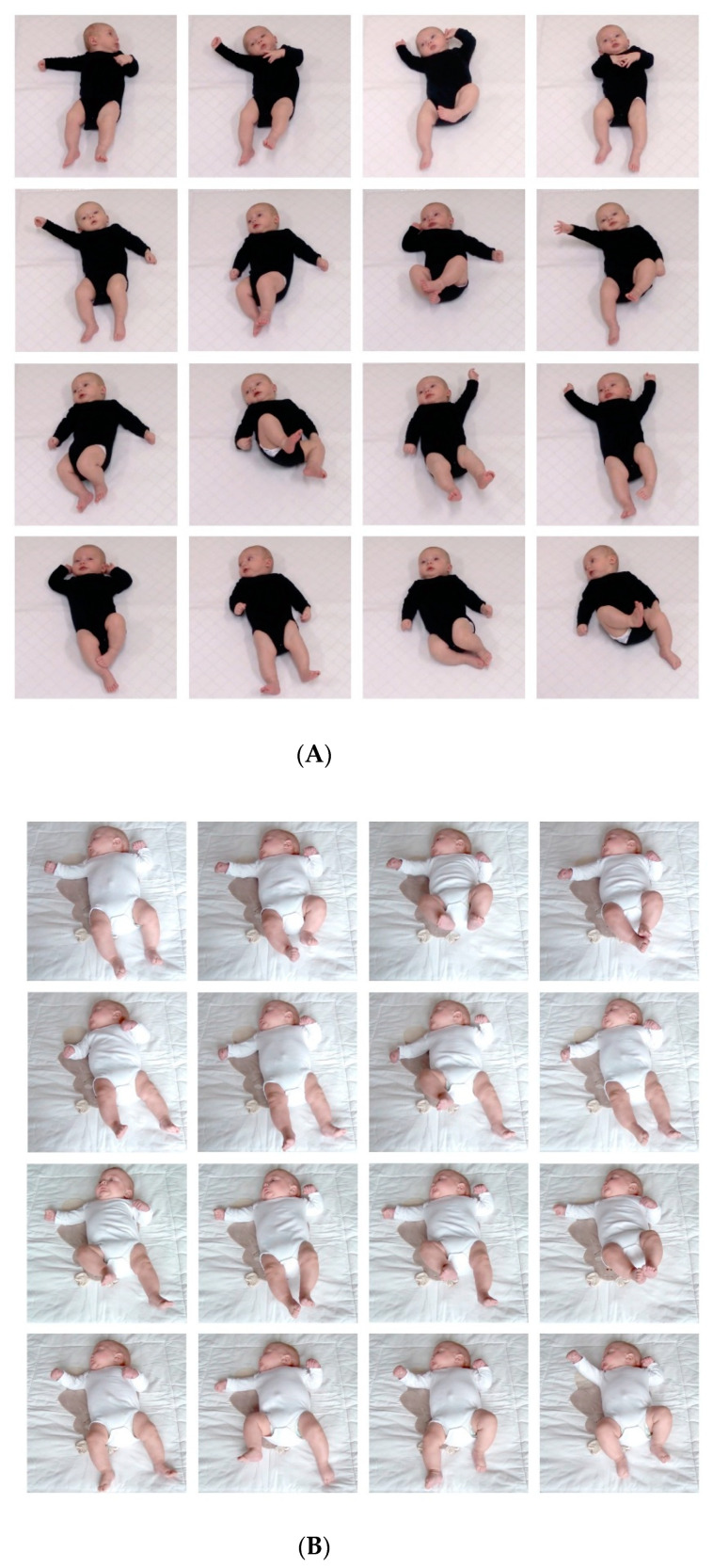
(**A**). Typical general movements characterized by movement complexity and variation in a 3-month-old infant. (**B**). Atypical general movements characterized by marked reduction in movement complexity and variation in a 3-months-old infant later diagnosed with bilateral spastic cerebral palsy (CP). Both subfigures consist of frames sampled from a video-recording of about 2 min. Figures produced with permission of the parents.

GMA consists of the evaluation of the quality of general movements (GMs), that is, spontaneous movements in which all parts of the body participate. GMs are the most frequently occurring movements in fetuses and young infants and can be observed until 4–5 months CA. GMA is based on a 3-min video recording of GMs in supine. It involves the evaluation of movement quality in terms of movement complexity and variation (the repertoire; Figure 2) and in terms of age specific characteristics, especially the presence of so-called fidgety movements in the last phase of GMs (2–5 months CA) [38,57,91]. GMA is a reliable technique, but it requires ample experience to become a reliable assessor [38,57]. GMA predicts CP very well, especially when performed in preterm infants in the fidgety phase (sensitivity 98%, specificity 91% [51]). Atypical GMs in the fidgety phase also have been associated with cognitive impairment and attention deficit and hyperactivity disorder at school age [38]. Retrospective data suggest that atypical GMs may also be associated with ASD [98]. It should be realized, however, that the predictive value of GMA in the general population has only been evaluated in one study. It revealed that atypical GMs predicted CP and serious neurodevelopmental disorder with sensitivities of 67% and 60%, respectively and specificities of 97% [49]. This means that GMA is an excellent tool to predict the young infant’s developmental outcome, especially in groups of at risk infants. The awareness that the technique requires extensive experience induced research on the possibilities of automated, marker-free GMA e.g., [99,100,101,102]. It is likely that automated GMA will become available within the next ten years.

The TIMP is—like GMA—a test for young infants. It is applicable from 34 weeks PMA to 16 weeks CA. The TIMP consists of 27 items based on spontaneous movements and 25 items based on elicited motor behavior observed in various infant positions [82,92]. The TIMP takes 20–40 min and has a good reliability [82]. Its predictive value for CP has not been reported. Studies on the predictive validity of the TIMP mainly included preterm infants. One study showed that neonatal TIMP scores predicted motor and cognitive development at 6 months CA with a sensitivities of 86% and 100% and specificities of 68% and 66%, respectively [103]. Long term prediction is best by TIMP assessments performed around 3 months CA, but the predictive values of the studies vary considerably. Low TIMP scores at 3 months have been associated with low AIMS scores at 12 months [104], but not with low AIMS scores at 15 months [105]. Peyton et al. [106] reported that low TIMP scores at 3 months predicted impaired motor, cognitive and language scores at 2 years with low sensitivities (41–57%) and high specificities (87–89%). Yet, Kolobe et al. [107] reported that low TIMP scores at 3 months predicted motor delay at 5.5 year with a sensitivity of 72% and a specificity of 91%.

The IMP is applicable in infants aged 3 to 18 months, or rather to the functional age at which infants have a few months of experience in independent walking [93,108]. It is a video based method evaluating gross and fine motor activities in various positions. It has four qualitative domains (variation, adaptability, symmetry and fluency) and one quantitative domain (performance, that is, motor milestones). Assessment and scoring of its 80 items take 25–30 min. The IMP is a reliable instrument [93]. Two retrospective clinical studies addressed IMPs prediction of CP. They showed that low IMP scores predicted CP well, one study reported areas under the receiver-operating characteristic curves: 0.89–0.99 [109], the other a sensitivity of 93% and a specificity of 81% [110]. Low IMP scores also have been associated with lower IQ scores at preschool and school age [111,112].

#### 3.3.3. Developmental Assessments

Developmental assessments evaluate the abilities of young children in various domains, for example, motor, cognitive, language and social domains. Examples of developmental assessments are the BSID [90], the Griffiths Scale of Child Development [113], the Mullen Scales of Early Learning [114] and SINDA’s developmental scale [88]. The developmental assessments are mainly used for discriminative purposes and monitoring of developmental progress. Developmental assessments are based on the notion that a delay in the achievement of multiple developmental milestones is associated with an increased risk of a neurodevelopmental disorder. Yet, developmental assessments in general only have a moderate capacity to predict neurodevelopmental outcome [82,115,116]. Nonetheless, application of SINDA’s developmental scale in an at risk population resulted in a prediction of intellectual disability at the age of 2 years with a sensitivity of 77% and a specificity of 92% [88].

The recommendations on early detection of CP by Novak et al. [52] indicated that the Developmental Assessment of Young Children (DAYC) [117] is a good instrument for the early detection of CP. DAYC is an interactive questionnaire for parents to report achieved milestones. However, only the 2013 publication described the association between low DAYC scores and CP in a high risk population. It did not report predictive values [117].

#### 3.3.4. Assessments Aiming at the Early Detection of ASD

Research has demonstrated that at the age of 6 months behavior of children later diagnosed with ASD is similar to that of typically developing children [50,118,119]. ASD’s signs of impaired social interaction and communication slowly emerge in the second half of the first postnatal year to become more evident in the beginning of the second year [50,119,120,121]. With increasing age the majority of children with ASD gradually loose social and communicative abilities that they had mastered previously [119]. Examples of specific early signs are deficits in joint attention, gazing at faces and orienting to their name being called [50,118,121]. An unspecific sign of ASD in infancy is atypical emotionality and self-regulation [88,122].

Of the multiple screening questionnaires available for the early detection of ASD, the Modified Checklist for Autism in Toddlers (M-CHAT) is most frequently used. The M-CHAT is designed for children aged 16 to 30 months. It consists of 23 parent-completed questions on behaviors that may signal ASD [123]. A recent systematic review and meta-analysis concluded that in children with developmental concerns M-CHAT has a sensitivity of 83% and a specificity of 51% to detect ASD [124]. To improve the predictive properties, M-CHAT developers have added the option of a structured follow-up telephone interview (M-CHAT-R/F) [125]. In low risk populations the follow-up interview is associated with better prediction, but a similar improvement is not present in high risk groups consisting of children with a sibling diagnosed with ASD (familial risk of ASD) [126]. Two large studies in the general population reported that M-CHAT-R/F has sensitivities of 33–39% and specificities of 95–98% to detect ASD [127,128]. One of them [127] indicated that sensitivity at 16–20 months was lower than that at 21–26 months. The combination of low sensitivity and high specificity values implies a low positive predictive value, that is, the presence of many false positives. Both studies reported however, that many of the clinicians involved had not performed the follow-up interview in case of a positive screen on the M-CHAT questionnaire, therewith missing the opportunity to reduce the number of false positives.

The Infant Toddler Checklist (ITC) is another screener for ASD. It deserves attention as its testing age ranges from 6 to 24 months. The ITC consists of 24 parent-completed questions addressing emotion, eye gaze, gestures and communication [129]. It has been studied less extensively than the M-CHAT. ITC’s ability to predict ASD emerges at 9 months and improves with increasing age [130]. A recent study applied the ITC longitudinally between 6 and 24 months in a group with a high proportion of children with familial risk of ASD. ITC had sensitivities of 55–77% and specificities of 42–85% to detect ASD. Prediction improved especially from 12 months onwards [131].

The above described studies indicate that the currently available screening instruments cannot be used to detect ASD in the general population, as they are associated with a high rate of false positives [132]. However, the screeners may be useful as a first step in the diagnostics in groups of children with parental concerns or familial risk, especially in children aged at least 12 months [124].

### 3.4. Summary: Early Diagnostics and the Developing Brain

The literature on early diagnostics of CP and intellectual disability focusses on detection in the first year, that of ASD on diagnostics in the second year post-term. Early detection of intellectual disability is mostly studied in groups at high risk of CP and predictive values are usually not specified for the two conditions separately. Therefore, I discuss below only early diagnostics of CP with or without intellectual disability and ASD.

Substantial evidence is available that in high risk infants—especially infants born preterm—early detection of CP is best with the combination MRI at TEA and GMA at 2–4 months CA [51]. Clinical assessments, including GMA, applied before this age have less predictive value than assessments with the same instruments performed at the age of at least 2–4 months. This age-dependent difference in predictive properties parallels the major transition in the developing brain occurring around 3 months. Around 3 months, the temporary fetal circuitries in the subplate in the primary motor and sensory cortices have been replaced by the permanent circuitries in the cortical plate. As a result, the functional sequelae of impairments in the permanent circuitries may first become expressed around 3 months, as prior to this age the transient circuitries, that may have been less affected, are still in charge of functional output, that is, neuromotor behavior.

In groups of high risk infants GMA is the clinical assessment with the best predictive properties to detect CP. However, GMA cannot be used in older infants, as GMs disappear when goal directed movements emerge. Beyond GMA-age, the instruments that have the best predictive properties are the HINE, SINDA and IMP. Of these three, HINE is the oldest instrument and—consequently—has been studied most. HINE is well able to predict CP and to a lesser extent intellectual disability. It has, however, the drawback that its criteria and cut-offs for ‘at risk’ are age-dependent and that the cut-offs are only available for a limited number of ages. The SINDA and IMP have been developed more recently. Of the two, especially SINDA deserves attention. Its neurological scale pairs simplicity (similar criteria and cut-offs for ‘at risk’ for all infant ages) with high predictive values for atypical neurodevelopmental outcome (CP and/or intellectual disability). In addition, its developmental scale has good predictive properties for intellectual disability. Finally, we should bear in mind that our knowledge on the predictive properties of the above discussed instruments in the general population is very limited. Only the study of Bouwstra et al. [49] assessed the predictive power of GMA at 3 months in the general population. It showed that GMA was able to detect CP and other serious neurodevelopmental disorders relatively well, but with lesser accuracy than is known for groups of high-risk infants.

The literature on ASD indicates that it is hard to detect ASD in the first postnatal year. The first signs of ASD slowly emerge in the second half of the first year, in particular from 9 months onwards [119,121]. The time of emergence corresponds to the age at which the temporal structures of the subplate in the association areas, including the prefrontal and the parieto-temporal regions, and the external granular layer in the cerebellum have disappeared. This implies that the permanent circuitries of the ‘social brain’ become first fully available from about 9 months onwards—the social brain that includes the medial prefrontal cortex, the inferior frontal gyrus, the insula, the inferior parietal lobule, the superior temporal gyrus, the fusiform gyrus, the cingulate gyrus, amygdala and cerebellum [133,134,135]. In other words, impairments in the networks of the social brain, including those associated with ASD [13,15,136], can be expressed first when these networks have become established. This may explain why the prediction of ASD first improves from 12 months onwards. During the second year, prediction of ASD with screening instruments is best in children with familial risk of ASD. This holds true in particular for the screener studied best, the M-CHAT-R/F. Prediction of ASD in the general population is less good and is associated with a high rate of false positives. The limited capacity of screening instruments to detect children with ASD, also in the second year of life, may be explained by the complexity of the social brain. It is conceivable that the social brain’s complexity and its associated protracted development is responsible for the heterogeneity in type [137] and timing of ASD symptomatology, therewith interfering with ASD’s early detection.

## 4. Early Intervention

This section briefly summarizes the approaches used in early intervention in young children with or at risk of developmental disorders and the evidence available on their effectiveness. During the last decades it has become crystal clear that the family is the cornerstone of early intervention [138,139,140,141]. Families are the pivotal environment of children, and family members are the key persons who may impact child development through daily interaction during caregiving and play [140]. How early intervention can be implemented best in family settings is a matter of debate [142]. Important questions that currently need to be answered are: (a) should family members function as co-therapists or should they stick to their role of parent and care-giver; and (b) should caregiver instruction and training be recommended, or rather caregiver coaching [143,144]?

In the next sections I summarize early intervention in (a) infants admitted to neonatal intensive care; (b) infants at low to moderate risk and (c) infants with or at (very) high risk of CP and intellectual disability; and (d) children with or at high risk of ASD.

### 4.1. Intervention in Infants Admitted to the Neonatal Intensive Care Unit

Research on early intervention in medically fragile infants admitted to neonatal intensive care has focused on infants born preterm. Over the years families have become increasingly involved in the care of their preterm newborns. Care evolved from family centered care, in which parents mainly had a supportive role and professionals provided most care, to family centered developmental care in which family members are important for care provision. Currently, family integrated care is getting implemented, a form of care in which parents provide all except the most advanced medical care for their infants [145,146,147]. In general, more parental involvement is associated with better outcomes of family and infant [148,149,150].

Medically fragile infants and their parents experience high levels of stress [148,149,150]. Early intervention, generally called developmental care, aims at the promotion of parent-infant interaction, stress reduction and provision of a supportive environment. Developmental care may be provided as the comprehensive program of Neonatal Individualized Developmental Care and Assessment Program (NIDCAP) [151,152] or by means of a selection of its components, such as coaching of parent-infant interaction and stimulation of kangaroo care, breast feeding, nesting and swaddling. In general, developmental care has small beneficial effect on the infant’s short-term neurodevelopmental outcome, but evidence is lacking that the effects persist after the age of 4 months CA [147,153].

### 4.2. Early Intervention in Infants at Low to Moderate Risk of CP and Intellectual Disability

Infants who have been critically ill in the neonatal period without having acquired a significant lesion of the brain are at low to moderate risk of developmental disorders [142]. For these infants a wealth of early intervention programs is available [154]. Effective programs focus on support and education of the caregivers, support of sensitive and responsive parent-infant interaction and stimulation of infant development. The programs’ effects have mainly been studied in preterm infants. The component ‘caregiver support and education’ has especially been associated with reduced maternal stress, anxiety and depression and improved maternal sensitivity and responsiveness [149,154]. A systematic review and meta-analysis indicated that the programs also have a beneficial effect on the child’s cognitive development up to and including preschool age and a minor effect on infant motor development [155]. In infants with a low to moderate risk NeuroDevelopmental Treatment (NDT) is not recommended [156,157].

### 4.3. Early Intervention in Infants with or at (Very) High Risk of CP and Intellectual Disability

Infants are considered at (very) high risk, of especially CP and intellectual disability, when early neuroimaging has indicated the presence of a significant brain lesion, such as periventricular leukomalacia, cortical infarction, periventricular-intraventricular hemorrhage complicated by post-hemorrhagic ventricular dilation or parenchymal hemorrhagic infarction [142]. Early intervention in infants at (very) high risk evolved from interventions focusing on the child’s motor development to programs that (a) involve the family; (b) address the child’s mobility, learning and knowledge and communication; and (c) focus on activities and participation of child and family and not on impairments such as deviant muscle tone or atypical reflexes [142]. Recently various programs have been developed and evaluated in (very) high risk infants, for example, Goals Activity Motor Enrichment (GAME) [158,159], the Small Step Program [160], Coping with and Caring for infants with special needs (COPCA) [94,157,161] and—for infants at high risk of unilateral CP—baby constraint-induced movement therapy (baby-CIMT) [162] and intensive bimanual therapy [163]. The programs agree that important components of early intervention in (very) high risk infants are: (a) family involvement; (b) goal oriented intervention; (c) challenges for the infant to explore and try out actions out by themselves, with trial-and-error learning; (d) minimization of so-called hands-on support; (e) application of the concept of environmental enrichment, that is, use a variety of toys, tasks and infant positions; and (f) implementation of assistive devices from early age onwards [142]. The programs have been associated with beneficial effects on the child’s motor and/or cognitive outcome [142]. Whether NDT is beneficial in children at (very) high risk is debated [142,164,165]. The debate may have its roots in the vast heterogeneity in the way that NDT is practiced, often not in line with the ideas of its developers [166]. Despite the fact that knowledge on early intervention in (very) high risk children has increased substantially in the last decade, multiple questions still need to be answered. The most urgent ones are the questions on the role of the family mentioned in the introduction of Section 4. Additional questions are: (a) how to achieve the optimal dosage of practice, as it is known that higher dosages are associated with better child outcomes, but also with worse family well-being [142]; and (b) which minimum of hands-on techniques is beneficial and when do hands-on techniques become counterproductive [165]?

### 4.4. Early Intervention in Infants with or at High Risk of ASD

A diversity of psychosocial intervention programs for young children with ASD has been developed. Pharmacological treatment of ASD is under investigation but is currently not applicable in clinical settings [167]. Parents play a central role in early psychosocial interventions, as the interventions are designed to incorporate learning opportunities in daily activities [141]. Families experience the interventions as demanding, as they are often performed for 20–40 h per week for periods up to two years [141,168]. Similar to the situation of early intervention in infants at (very) high risk of CP and intellectual disability, future studies need to address the question of optimal dosage of intervention, that is, the search of the optimal balance between child development and family well-being.

The most frequently used approaches of psychosocial intervention are: (a) behavioral approaches that are based on operant learning; they are characterized by discrete stimulus presentation, prompted exhibition of the desired response and provision of positive feedback; (b) developmental approaches that use the child’s developmental drive for exploration to improve parent-child interaction, including joint attention and synchrony in interaction; (c) interventions that combine the behavioral and developmental approach [169,170]. The large majority of studies on the effect of early intervention has been performed in children diagnosed with ASD, implying that the children’s average age was at least 2.5 years of age. Recent systematic reviews on children of at least 2.5 years concluded that most studies suffered from a high risk of bias [169,170,171,172,173]. This was particularly true for the studies evaluating behavioral approaches. Consequentially, their reports of a beneficial intervention effect on the child’s adaptive behavior and cognition should be interpreted with caution [170,171,173]. The limited evidence available on the effect of early intervention in ASD indicates that interventions using a developmental or a combined developmental and behavioral approach may have a positive effect on the child’s play and social communication [170,172].

It is unclear whether the age at intervention affects outcome. Granpeesheh et al. indicated that their behavioral intervention was only associated with better outcomes in children younger than 7.2 years [174]. This may suggest that the window of optimal intervention opportunities closes at around 7 years, similar to the window of plasticity described for interventions in amblyopia [175]. Fuller and Kaiser [172] assessed the effect of age in younger children. They reported that the effect of age on the intervention’s effectiveness on social communication followed an inverted U-curve. The optimum age for intervention was 3.8 years [172], that is, the age at which social cognition has developed so far that children start to become group-minded and therewith start to take an objective perspective on things [176].

Few studies addressed the effect of early intervention in infants at familial risk of ASD. French and Kennedy [169] reviewed the three randomized control trials available and concluded that only one had a relatively low risk of bias. The latter study [177] evaluated the effect of 12 sessions of the iBASIS-VIPP (British Autism Study of Infant Siblings—Video Interaction for Promoting Positive Parenting) program—a parent-mediated social communication intervention—against no intervention in 54 infants at familial risk of ASD. The intervention was provided when the children aged 9 to 14 months. The data indicated that at the end of the intervention, iBASIS-VIPP was associated with improved attentiveness of the infant to the parent, improved adaptive behavior, improved attention disengagement, fewer autism-risk behaviors and increased non-directiveness of the parents. Yet, it should be noted that only the effect on parent behavior reached statistical significance [177]. Follow-up at the age of 3 years indicated the presence of similar effect trends: the intervention was associated with less autism prodromal symptoms, better attention and social communication and better parent responsiveness and synchrony during interaction with the child [178]. It should be noted, however, that of the 53 participants in the follow-up study only six had been diagnosed with ASD, whereas another 15 showed atypical behavioral characteristics but no ASD.

Overall, this means—as the recent systematic reviews concluded [169,170,171,172,173]—that studies on the effect of early intervention in ASD with good methodological quality are urgently needed. For the time being, interventions with a developmental approach with or without behavioral components are most promising to have a beneficial effect on social communication.

## 5. Conclusions

Early detection of developmental disorders is challenging. The challenge is inherent to the developmental characteristics of the young brain. Neuroimaging is certainly helpful in early diagnostics, especially in infants who start post-natal life in neonatal intensive care. The large majority of children start life differently; in these children, early diagnostics has to rely on the functional expressions of brain activity, that is, behavior that may be observed in daily life or clinical settings. The ubiquitously used developmental milestones are helpful but have limited predictive value. Early detection of CP with or without intellectual disability is best with GMA, HINE and SINDA, especially from 2–4 months onwards. A caveat is that little information is available on the predictive properties of these instruments in the general population. Early detection of ASD is difficult. The best validated results have been reported for the MCHAT and—in younger children—the ITC, especially when applied in children with familial risk of ASD aged at least 12 months.

The data indicate that early detection of developmental disorders improves substantially when the transient structures of the young brain have been taken over by permanent circuitries. One transition is at 2–4 months, when the cortical subplate in the primary motor and sensory cortices has dissolved—hence the improved prediction of CP with or without intellectual disability from this age onwards. A second transition occurs around 12 months when the cortical subplate in the prefrontal and parieto-temporal cortices and the external granular layer in the cerebellum have disappeared. After this transition prediction of ASD becomes possible.

We gradually understood the pivotal role of the family in early intervention, as the family forms the main environment of children. Currently, early intervention aspires to guide the family as the primary unit of intervention, implying that it aims at promoting activities and participation of child and family. Early intervention in medically fragile newborns focuses on the family and developmental care of the infant. Evidence indicates that it improves family well-being and infant outcomes up to the age of 4 months CA. This implies that the infant effect does not extend to the period of life after the major transition at 2–4 months. Ample evidence is available that early intervention in infants at low to moderate risk of CP and intellectual disability is successful in promoting infant and family outcomes. Relatively little evidence is available on the effect of early intervention in young children at (very) high risk of CP, intellectual disability or ASD. The available evidence suggests that skills improve, when they are practiced intensively in a playful daily care giving setting. This suggests that interventions in which families are coached on the nature of the skills emerging and the ways in which the child may explore and perform these skills are most promising. In other words, interventions that are tailored to the family, the child and their interaction, may be most successful.

## Data Availability

Not applicable.
